# Telehealth and Speech Therapy: usability assessment of an orientation and prevention program of orofacial myofunctional changes, during the COVID-19 pandemic

**DOI:** 10.1590/2317-1782/20232022057en

**Published:** 2023-05-29

**Authors:** Amanda de Siqueira Cabral, Raíssa Gomes Magalhães, Giovanna Régis Viana, Nataly Santana de Araújo, Stephanie Ribeiro Silva, Melissa Picinato-Pirola

**Affiliations:** 1 Faculdade de Ceilândia, Universidade de Brasília – UnB - Brasília (DF), Brasil.

**Keywords:** Health Education, Health Promotion, Social Isolation, Speech, Language and Hearing Sciences, Telemedicine, Educação em Saúde, Promoção da Saúde, Isolamento Social, Fonoaudiologia, Telessaúde

## Abstract

**Purpose:**

To assess the effect of an virtual speech-language orientation program, as well as the prevention of orofacial myofunctional alterations.

**Methods:**

Fifty-five volunteer residents aged between 18 and 50 years of age residents of Federal District participated in the study, 14 men and 41 women with an average of 28. The orientation program was divided into five stages (1) The preparation of material to be used in the orientation program, (2) The completion of a semi-structured questionnaire made available through Google Forms, (3) Completion of a pre-orientation program questionnaire, (4) utilization of the speech therapy orientation program, (5) Completion of the post-orientation program questionnaire. To analyze the results the McNemar statistical test was used considering the absolute frequency (N), enabling comparison through a paired sample. The significance level adopted was 5%.

**Results:**

Statistically significant differences were seen in 10 of the 19 questions asked in the pre and post-orientation program questionnaires, proving the effect of the orientation program and improvement in participants’ knowledge. In addition the participants were satisfied with the program and the content.

**Conclusion:**

The orientation program focused on health promotion and prevention of orofacial myofunctional alterations and combined with telehealth brought significant changes to the reality of the participants, favoring the quality of life of these individuals and changing their reality.

## RESUMO

**Objetivo:**

Verificar o efeito de um programa de orientação fonoaudiológica virtual, bem como prevenir alterações miofuncionais orofaciais.

**Método:**

Participaram deste estudo 55 voluntários moradores do Distrito Federal com idade entre 18 e 50 anos, sendo 14 homens e 41 mulheres, com idade média de 28 anos. O programa de orientação proposto foi dividido em cinco etapas: 1) elaboração dos materiais a serem utilizados no programa de orientação; 2) preenchimento de questionário semiestruturado por meio da plataforma Google Forms; 3) aplicação do questionário pré-programa de orientação; 4) realização do programa de orientação fonoaudiológica; 5) preenchimento do questionário pós-programa de orientação. Para a análise dos resultados utilizou-se o teste estatístico McNemar considerando a frequência absoluta (N), possibilitando a comparação por meio de amostra pareada. O nível de significância adotado foi de 5%.

**Resultados:**

Observou-se diferenças estatisticamente significativas em 10 das 19 questões propostas nos questionários pré e pós-programa de orientação, evidenciando o efeito da orientação proposta e aquisição de conhecimentos por parte dos voluntários. Além disso, os participantes demonstraram satisfação em relação ao programa e aos conteúdos disseminados.

**Conclusão:**

O programa de orientação com enfoque na promoção da saúde e prevenção de alterações miofuncionais orofaciais aliado à telessaúde trouxe mudanças significativas para a realidade dos participantes, favorecendo a qualidade de vida desses indivíduos e tornando-os agentes transformadores de realidade.

## INTRODUCTION

Throughout life we adopt various habits and types of behavior according to our experiences and our body's requirements. Among these are oral habits, which are defined as learned neuromuscular habits that become unconscious^(1)^. On the other hand when there is a change in the organism, be it structural, morphological or psychological, harmful patterns may arise called deleterious habits^(1)^. Some of the more common habits such as pacifiers and/or bottle usage, finger sucking, onychophagy (nail biting), atypical tongue pressing during speech, lip sucking, mouth breathing, bruxism and object-biting habits^(2,3)^, can become harmful in the long term due to extended duration, high frequency and intensity of the habit resulting in overloading of the stomatognathic system (SE)^(1,4)^.

Deleterious oral habits can cause changes in orofacial functions, such as chewing, swallowing, breathing and speech, in addition to changes in the temporomandibular joint (TMJ), resulting in a temporomandibular disorder (TMD), especially during increased periods of apprehension and anxiety^(5)^.

The emergence of the coronavirus pandemic and the requirements for social distancing and self isolation to help halt the advance of the virus^(6)^ resulted in a growth of psychological disorders and consequently an increase in anxiety, depression and post-traumatic stress^(7)^. These disorders can lead to an increase in deleterious oral habits and consequently changes in orofacial functions^(1,4)^.

Social isolation can lead to changes in sleep patterns caused by a lack of daily routines, which can change sleeping and waking times, and in the long term these changes in sleep patterns can provoke sleep disorders, consequently care is needed in this area to ensure essential and proper bodily function^(8)^.

Telehealth has emerged as an effective way of distributing health-related services and information during the pandemic and allows for long-distance consultations, care and disease prevention measures^(9)^. Speech-language pathology and audiology, resolution No. 580 of August 20, 2020 established guidelines for carrying out teleservices and the use of services such as tele-education^(10)^.

Social isolation is a factor which can contribute to changes or the worsening of habits that impair adequate performance of orofacial functions and these are related to a person’s quality of life. Health promotion and education have the purpose of promoting progress and social transformation so that individuals become active members in the process of health improvement.

The objective of this study was to verify the effect of a virtual speech therapy program, as well as to prevent orofacial myofunctional alterations.

## METHODS

This study was approved by the Research Ethics Committee of Faculdade de Ceilândia – Universidade de Brasília, protocol number 4.341.780.

This is an observational, cross-sectional and quantitative carried out in 2021 during the COVID-19 pandemic with residents of the District Federal, who were required to have access to WhatsApp (A cross-platform centralized instant messaging and voice-over-IP service) and as well as be aged between 18 and 50 years old. The following participants were excluded from the research, speech-language therapists and students of speech-language pathology courses, as well as participants who did not have internet access and who did not complete all the stages of the study. In addition when considering the questionnaires used for the study, participants with literacy problems were also were excluded from the research. All the volunteers agreed to participate in the research by signing a consent form (FICT).

To prepare the materials used in this research, a bibliographic search was carried out of scientific literature in the PubMed and Scielo databases, according to each theme, as shown in Table 1. The searches were based on a predefined script.

**Table 1:** Bibliographic survey according to each theme for the design of the questionnaire and the orientation program

**Theme**	**Key Words**	**Number of articles found**	**Number of articles selected**
Sleep	Sleep, Sleep Deprivation, Sleep Hygiene, Multidisciplinary team, Adolescent, Young Adult, Adult, Social isolation, Quarantine.	529	88
Breathing	Breathing, Mouth Breathing, Speech Therapy, Definition.	109	47
Chewing	Chewing, Masticatory Muscles, Stomatognathic System, Stomatognathic System Abnormalities, Myofunctional Therapy, speech therapy, Speech-Language Pathology.	145	30
TMD	Speech Therapy, Temporomandibular Joint Disorders, Temporomandibular Joint (TMJ) Dysfunction Syndrome, Primary Prevention, Anxiety, Hearing.	674	119
Speech and oral habits	Speech, Speech Therapy, habits, deleterious, harmful, oral, speech disorders, bruxism.	553	41

Caption: TMD = Temporomandibular disorders

1. Definition

2. Etiology

3. Classification

4. Diagnosis and treatment

5. Professionals involved in the treatment

6. Preventive Measures

For each theme 3 types of learning material were made available, in the following order. A video of a maximum duration of 10 minutes containing the definition, etiology and consequence of each habit or behavior. A PDF booklet pointing out the main points of each subject, prevention measures and professionals involved in the treatment of orofacial myofunctional alterations, in addition to specific topics on each subject and a short video covering trivia about the topic of the day, called “Minute Health”. At the end of the second day of each topic a specific time was chosen for the participants to ask question or resolve any queries (Table 2).

**Table 2:** Distribution of the orientation program

Day	Theme	Type of material	Content
1	Sleep	Video	Definition and function; Sleep physiology (normal versus altered sleep); Main sleep disorders.
2	Sleep	Guidebook and Minute Health (Short Video)	Professionals involved in diagnosis and treatment; Step by step of healthy sleep; Applications to monitor sleep and the immune system.
3	Breathing	Video	Definition physiological process altered breathing; Etiology, Consequences of oral or oronasal breathing; Association between mouth breathing and other orofacial functions.
4	Chewing	Guidebook and Minute Health (Short Video)	Professionals involved in the treatment; Step by step of nasal hygiene; Preventive measures for mouth breathing.
5	Chewing	Video	Definition of chewing; Types of chewing; Factors that interfere in the masticatory pattern; Chewing steps.
6	Chewing	Guidebook and Minute Health (Short Video)	Tips for improving chewing and highlighting the importance of effective chewing; Difference between food consistencies; Professionals involved in the treatment; Relationship between weight loss and chewing.
7	TMD	Video	Definition of the temporomandibular joint (TMJ); Definition of Temporomandibular Disorder (TMD); TMD symptomatology; Morphological and muscular alterations caused by TMD.
8	TMD	Guidebook and Minute Health (Short Video)	TMD diagnosis and treatment; Professionals involved in the treatment; TMD preventive measures; Relationship between anxiety, Stress and ATM; Relationship between TMD and hearing.
9	speech/oral habits	Video	Definition of deleterious oral habits; Frequent deleterious habits and their consequences; Speech definition; Differentiation between voice, chartspeech and language; Most common speech disorders.
10	speech/oral habits	Guidebook and Minute Health (Short Video)	Professionals involved in the treatment; Measures to prevent harmful oral habits; Tips for improving speech.

Caption: TMD = Temporomandibular disorders

For the recruitment of participants, an initial semi-structured questionnaire was published on social networks such as Instagram, WhatsApp and Facebook and made available on Google forms and prepared according to the content of the speech therapy program and comprised of sociodemographic questions for the purposes of sample characterization and identification of participants, in addition to the FICT and their WhatsApp contact details. Then, 223 individuals were contacted through their WhatsApp number which they provided in the initial questionnaire, inviting them to participate in the speech-language pathology orientation program, as well as providing the access link to the pre-test questionnaire speech therapy program.

After completing this stage, only 55 volunteers accessed the pre-program questionnaire, and were included in the WhatsApp groups to receive the material prepared for the speech-language pathology guidance program. The 55 volunteers were divided into two groups just to highlight that they were allocated into two different groups in WhatsApp. Group A consisted of 7 men and 22 women, totaling 29 participants and group B consisted of 7 men and 19 women, totaling 26 participants. The average age remained at 28 for both groups. A total of ten days was given to each group to complete the orientation program of which two days were allocated to each subject.

When considering the level of education of the participants in this research, there was a higher frequency of participants who were graduating (46.1%), followed by those who had completed higher education (38.5%) and others who had completed high school (15.4%).

The questionnaire contained 21 questions, of which 18 questions about the contents that would be covered in the proposed orientation program, which were performed again at the end of the program for the purpose of comparing the answers after the program, and a further 3 questions regarding the degree of satisfaction of the participants with the orientation program.

After completing the speech therapy orientation program, the post-program questionnaire was given to the participants, this questionnaire contained the same questions as the pre-program questionnaire and additionally contained questions about satisfaction with the program (Chart 1).

**Chart 1:** Pre-program and post-program questionnaire A

Q1. Do you think that sleep deprivation can bring about changes such as tiredness, irritation, concentration and memory difficulties, in addition to feeling very sleepy during the day?
() Yes () No () Don’t know
Q2. Do you agree that the treatment of Obstructive Sleep Apnea (OSA) involves lifestyle changes, such as regular physical exercise and nutritional education?

() Yes () No () Don’t know
Q3. Do you think that the speech therapist can help in the treatment of OSA?

() Yes () No () Don’t know
Q4. Do you consider that ideal breathing should be performed by:

() Mouth () Nose () Nose and mouth () Don’t know
Q5. Do you know how nasal cleaning is performed?

() Yes () No () Don’t know
Q6. Which professionals are involved in the treatment of mouth breathing?
() Speech therapist () Nutritionist () Neurologist () Dentist () Otorhinolaryngologist
Q7. Do you think there is a proper way to chew?
() Yes () No () Don’t know
Q8. Do you think that chewing can interfere with facial muscles?
() Yes () No () Don’t know
Q9.Do you think that the speech therapist can help in the treatment of chewing disorders?
() Yes () No () Don’t know
Q10 Earache, pain and popping when opening/closing your mouth and pain when chewing can be symptoms of:
() Temporomandibular Disorder (TMD) () Migraine () Stress () Muscle weakness () Don't know
Q11. Do you think that stress and anxiety can cause pain and muscle tension in the temporomandibular joint region (TMJ)?
() Yes () No () Don’t know
Q12. Do you believe that grinding or clenching your teeth can cause temporomandibular disorders (TMD)?
() Yes () No () Don’t know
Q13. Do you think that TMD treatment needs a multidisciplinary team that includes a speech therapist?
() Yes () No () Don’t know
Q14. Do you think that oral habits (pacifier/bottle usage, nail biting, biting objects) can be harmful?
() Yes () No () Don’t know
Q15. Do you think that oral habits can influence breathing?
() Yes () No () Don’t know
Q16. Do you think that oral habits can influence chewing?
() Yes () No () Don’t know
Q17.Can the use of a pacifier, bottle and thumb sucking cause speech disorders?
() Yes () No () Don’t know
Q18. Is there a difference between voice, speech and language?
() Yes () No () Don’t know
Q19. How satisfied were you with the program?
() Not satisfied () Somewhat satisfied () Indifferen () Satisfied () Very satisfied
Q20. How likely are you to use the tips and information learned from the program in your routine? 1 being unlikely and 5 very likely.
() 1 () 2 () 3 () 4 () 5
Q21. Leave any suggestions, compliments and criticisms here*

* Questions asked only in the post-program questionnaire

Caption: Q = question; TMD: temporomandibular disorders

For statistical analysis of the questions, the McNemar test^(11)^ was used, considering the absolute frequency (N), with the answers “no” and “don't know” being grouped together. The significance level was set at 5%. The descriptive questions were considered invalid for this test, for analysis the answers were categorized and gathered according to the main message of each answer given by the participants.

## RESULTS

After completing the program and statistical analysis of the absolute and relative frequencies (Table 3), it was found that in 10 out of the 19 comparable questions there was a significant difference (p ≤0.05). It is also worth noting that question 1 was not suitable for this statistical test because the distributions of values in the pre and post questionnaires were statistically equal, that is all the participants marked the alternative “yes” before and after the speech therapy orientation program.

**Table 3:** Comparison of participants' answers in the pre- and post-program questionnaire of the speech therapy program

		Pre-program		Post-program		P-value
		N	%	N	%	
Q2	Yes No/Don’t know	41	75.0	54	98.0	0.002*
		14	25.0	1	2.0	
Q3	Yes No/Don’t know	41	75.0	54	98.0	0.0009*
		14	25.0	1	2.0	
Q5	Yes No/Don’t know	12	22.0	47	85.0	<0.001*
		43	78.0	8	15.0	
Q7	Yes No/Don’t know	48	87.0	55	100.0	0.0156*
		7	13.0	0	0.0	
Q8	Yes No/Don’t know	50	91.0	53	96.0	0.6875
		5	9.0	2	4.0	
Q9	Yes No/Don’t know	47	85.0	53	96.0	0.0703
		8	15.0	2	4.0	
Q11	Yes No/Don’t know	49	89.0	53	96.0	0.3750
		6	11.0	2	4.0	
Q12	Yes No/Don’t know	42	76.0	52	95.0	0.0009*
		13	24.0	3	5.0	
Q13	Yes No/Don’t know	42	76.0	54	98.0	0.0039*
		13	24.0	1	2.0	
Q14	Yes No/Don’t know	54	98.0	55	100.0	1.0000
		1	2.0	0	0.0	
Q15	Yes No/Don’t know	45	82.0	55	100.0	0.0019*
		10	18.0	0	0.0	
Q16	Yes No/Don’t know	49	89.0	55	100.0	0.0312*
		6	11.0	0	0.0	
Q17	Yes No/Don’t know	37	67.0	51	93.0	0.0500*
		18	33.0	4	7.0	
Q18	Yes No/Don’t know	50	91.0	55	100.0	0.0009*
		5	9.0	0	0.0	

Caption: Q = question; N = Absolute Value; % = Relative Value

**Figure 1:**
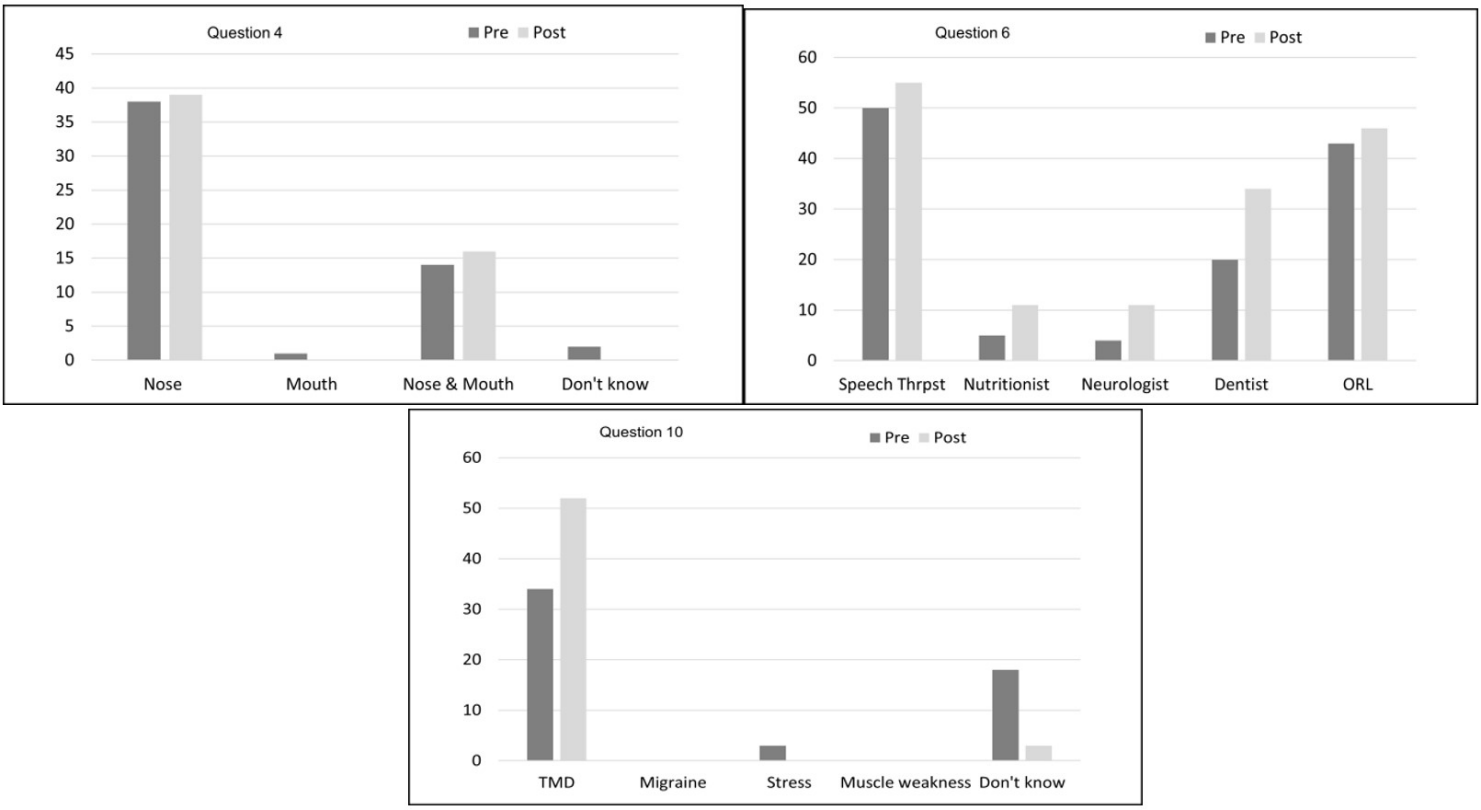
Comparison between the answers to questions 4, 6 and 10 of the pre and post-program questionnaires. (Q4) Adequate structure for breathing. (Q6) Professionals involved in the treatment of mouth breathing. (Q10) Possible cause of earache, pain and popping when opening/closing the mouth and pain when chewing

Question four about ideal breathing (Q4), had a higher frequency of the alternative “nose”, followed by the “nose and mouth” alternative in both questionnaires, which showed a similar frequency for all alternatives in the comparison between the pre- and post-orientation program questionnaires (Figure 1).

Caption: ORL = otorhinolaryngologist; TMD = temporomandibular disorder

When asked about the multidisciplinary team involved in the treatment of mouth breathing, the speech therapist, otolaryngologist and the dentist were the most mentioned professionals, respectively. In addition in the post-program questionnaire, there was an increase in the mention of these professionals, with emphasis on the dentist, which increased by 41% (Figure 1).

In question 10 (Figure 1) there were no answers to the alternatives “migraine” and “muscle weakness”. There was also an increase in the correct alternative “TMD” in the post-program questionnaire, associated with a decrease in the frequency of “stress” and “don't know” answers.

The orientation program satisfaction questions (Q19 to Q21), 65% of the participants were very satisfied, 33% satisfied and 2% not very satisfied. When asked about the guidelines provided in the program and their daily lives, 60% reported “very likely”, 29% answered “likely” and 11% indicated “unlikely”.

The last question (Q21) gave the participants the opportunity to express their opinions about the orientation program. Forty participants praised the program in terms of organization, quality of materials and content, preparation by the researchers, how queries and doubts were handled and finally didactics and reception.

## DISCUSSION

Prevention is the best way to avoid prejudice occurring. In this context, the guidance program in this study aimed to prevent orofacial myofunctional changes resulting from harmful oral habits and other such behavior, which increased significantly due to COVID-19 social isolation measures.

This type of program disseminates fundamental information for the prevention of diseases. The study by Guimarães and Picinato-Pirola^(12)^ carried out an educational program in schools aimed at preventing and improving knowledge about mouth breathing and its causes and consequences with an objective and results similar to this study. This reaffirms the relevance of speech therapy orientation programs.

This study used telehealth as the method for health promotion through awareness and dissemination of information about the proposed themes. In addition the technological resources used such as videos, pdf booklets and WhatsApp, were based on information and communication technology^(13,14)^, which reaffirms that multimedia can be used successfully as a learning medium.

Considering the questions about sleep (Q2 and Q3), there were statistically significant differences when comparing the pre and post questionnaires. We noted that the study audience already had prior knowledge about the need for lifestyle changes in cases of Obstructive Sleep Apnea (OSA), this data corroborates studies^(15,16)^ that investigated the prevalence of sleep alterations in the Brazilian population, emphasizing that those who exercise minimize the chances of suffering from sleep disorders. In addition, a team is needed to treat this condition (Q3), so the participants indicated that speech therapists should be an integral part of this team and can perform myofunctional therapy to adjust muscle tension, mobility and posture of organs phonoarticulatory organs, decrease apnea and hypopnea indexes, the awakening index, excessive daytime sleepiness, as well as improve sleep quality and quality of life^(17)^.

Q4 introduces the subject of breathing, highlighting the structure for the execution of this vital function. The nose is considered the ideal organ for breathing due to its’ structures which are capable of filtering, heating and humidifying air^(12,18)^, the participants already knew about about this information (Figure 1). However, in the pre-program questionnaire (Q5) the participants reported that they didn’t know about the procedure for performing nasal cleaning, a practice that significantly influences nasal aeration^(19)^, however, after the program there was a 63% increase in knowledge about this.

In cases of treatment of mouth breathers, a multidisciplinary team is required, which must consist of a speech therapist, otorhinolaryngologist and dentist, among others. However most people are unaware of the professionals who should carry out the treatment in these cases, but with guidance this can be changed, as demonstrated in the study by Guimarães and Picinato-Pirola^(12)^, which showed an increase in the mention of speech therapists, otorhinolaryngologists and dentists after an orientation program (Q6). In this study, the same thing happened, with a 41% increase in the citing of dentists.

Regarding chewing (Q7 to Q9), it is a well known fact that the ideal masticatory pattern is the alternating bilateral one and a preference for a different form of mastication, can impair the function with possible consequences of TMD and an asymmetrical face^(20,21)^. When asked we found that the participants learned about this after the speech-language pathology orientation program. However, if the individual has a chewing preference, there may be an alteration in the facial muscles, requiring an intervention to adjust mobility and muscle tension, which may be carried out by the speech therapist^(21)^. The participants showed they already knew about this subject.

TMD can cause symptoms such as earache, pain and popping of the ears when opening and closing the mouth and pain when chewing, however quite often an individual with these symptoms may not be aware of this dysfunction as demonstrated by the results of the pre-program questionnaire (Q10), it is extremely important to promote health in Speech-Language Pathology and Audiology about issues that can be prevented, making society able to identify the symptoms of this change and to seek specialized help.

The speech therapist is an integral member of the multidisciplinary team and is responsible for the treatment of TMD. The role of the speech therapist is crucial in carrying out myofunctional therapy (OMT) and utilizes resources such as laser therapy to adapt orofacial structures, as well as balance the orofacial functions^(22)^. This information was successfully conveyed to the participants (Q13) as seen by analyzing the statistical analysis.

The presence of deleterious oral habits can be harmful due to their long duration, high frequency and intensity, which can overload the stomatognathic system^(1,4)^. The structures that make up this system work together in a balanced and controlled manner and are responsible for the vital functions of breathing, sucking, chewing and swallowing, in addition to speech. Consequently if there is a change in these structures, the functions can also undergo changes^(2,3)^, and as a way of preserving these structures, prevention is considered in order to provide the balance of the stomatognathic system through awareness of the harm that can be caused by harmful habits^(2,3)^. This issue was addressed during the program developed in this study and comparing the pre and post-program responses (Q14 to Q17), some participants' already knew of the damage caused by harmful oral habits, specifically the use of pacifiers, bottle feeding and onychophagy, chewing, breathing and speaking. When studying the answers in the post questionnaire 100% of the answers in Q15 and 16 were correct, as well as a 26% increase in the post-program answers of Q17, which confirms acquisition of knowledge of this topic in the program.

The last question subject to statistical analysis using the test adopted in this study refers to the difference between speech, voice and language. There was a significant increase in the answers referring to the “yes” alternative, which shows the 100% effectiveness of the content in the post-program questionnaire. This result can be justified by the type of material used to disseminate this content. It was a short video, therefore it does not require a greater attentional focus to understand the information, enhancing the transmission of knowledge^(23)^.

The groups in this study were satisfied with the information in the orientation program (Q19 to Q21) and with the quality of material and content, handling of queries, didactics and reception being pointed out as factors that potentiated the propagation of the orientations.

This study demonstrated positive results about the telehealth orientation, and it was possible to see that the program proved to be effective and fulfilled its purpose of educating, as well as informing the adult population of the Federal District, corroborating the results found in another study^(12)^.

Among Speech-Language Pathology research, there are still very few studies that aim to promote health and prevent orofacial myofunctional alterations, deleterious oral habits and improve sleep behavior, so the purpose of this research was to encourage such actions in order to promote and increase studies on the theme.

It is worth mentioning that the limitations of this study were the low adherence of the participants and the lack of control over the studies of the program content by the participants. A further orientation program is recommended in a controlled access virtual environment.

## CONCLUSION

In view of the findings in this research, the orientation program focused on health promotion and prevention of orofacial myofunctional alterations combined with telehealth bringing significant changes to the reality of the research participants during a critical period of the pandemic and helped to minimize the conditions that favor and intensify orofacial myofunctional changes, making it possible to use the autonomy of individuals to disseminate information and guidelines that are capable of transforming reality and favoring a better quality of life using technological resources associated with health.
